# Mental pain and depressive symptoms in the determination of suicidal ideation among psychiatric patients

**DOI:** 10.1192/j.eurpsy.2023.1063

**Published:** 2023-07-19

**Authors:** E. Rogante, S. Sarubbi, D. Erbuto, M. A. Trocchia, S. P. Iancau, F. Manfreda, F. Splendori, M. Tinè, A. V. Vallerga, L. Longhini, I. Berardelli, M. Innamorati, M. Pompili

**Affiliations:** 1 Department of Human Neurosciences; 2 Department of Neurosciences, Mental Health and Sensory Organs, Suicide Prevention Centre, Sant’Andrea Hospital; 3 Psychiatry Residency Training Program, Psychiatry Unit, Sant’Andrea Hospital, Sapienza University of Rome; 4 Department of Human Sciences, European University of Rome, Rome, Italy

## Abstract

**Introduction:**

Though the literature suggests a strong association between depressive symptoms and suicidal ideation, in clinical practice, it is often observed that many patients who show those symptoms, even the most severe, do not experience suicidal ideation. Thus, the association between depressive symptoms and suicidal ideation is insufficient to explain the complexity behind suicide. From Shneidman’s point of view, the common feature in patients with suicidal ideation and suicidal behavior seems to be mental pain, defined by the author as “psychache” and characterized by a distressed state of mind, in which the subject experiences extreme angst, hopelessness and in which pain is seen as unsolvable. Individuals with depressive symptoms are suicidal only when psychache is so unbearable that suicide is perceived as the only option.

**Objectives:**

Our study aimed to investigate the association among depressive symptoms, mental pain, and recent suicidal ideation, specifically whether mental pain could mediate the relationship between depressive symptoms and current suicidal ideation in a sample of psychiatric patients.

**Methods:**

Participants were 206 adult patients (49.5% females). Patients were assessed for psychiatric diagnoses according to DSM-5. For the study, the following instruments were administered: the Columbia-Suicide Severity Rating Scale (C-SSRS), the Beck Depression Inventory-2 (BDI-2), and the Orbach & Mikulincer Mental Pain Questionnaire (OMMP).

**Results:**

32.5% of the patients had bipolar disorder, 21.4% had MDD, 24.8% had schizophrenia or other psychotic disorders, and 15% had a personality disorder. About 34% of the patients reported recent suicidal ideation with at least some intent to act. Recent suicidal ideation was associated with both mental pain and depressive symptoms, but mental pain completely mediated the association between depression and suicidal ideation (β=.04, SE=.01, 95% CI (.01/.06).

**Conclusions:**

Our study indicated that patients with more severe depressive symptoms are more likely to report suicidal ideation and that the presence of mental pain could explain this association thoroughly. Thus, in clinical practice, the identification of mental pain confirms its crucial role in the assessment of suicide risk and in the understanding of the individual’s unique pain.

**Disclosure of Interest:**

None Declared

